# segment_liftover : a Python tool to convert segments between genome assemblies

**DOI:** 10.12688/f1000research.14148.2

**Published:** 2018-06-08

**Authors:** Bo Gao, Qingyao Huang, Michael Baudis

**Affiliations:** 1 Institute of molecular Life Sciences, University of Zürich, Zürich, CH-8057, Switzerland; 2 Swiss Institute of Bioinformatics, University of Zürich, Zürich, CH-8057, Switzerland

**Keywords:** Genome assembly, liftover, remap, copy number segment.

## Abstract

The process of assembling a species’ reference genome may be performed in a number of iterations, with subsequent genome assemblies differing in the coordinates of mapped elements. The conversion of genome coordinates between different assemblies is required for many integrative and comparative studies. While currently a number of bioinformatics tools are available to accomplish this task, most of them are tailored towards the conversion of single genome coordinates. When converting the boundary positions of segments spanning larger genome regions, segments may be mapped into smaller sub-segments if the original segment’s continuity is disrupted in the target assembly. Such a conversion may lead to a relevant degree of data loss in some circumstances such as copy number variation (CNV) analysis, where the quantitative representation of a genomic region takes precedence over base-specific accuracy. *segment_liftover* aims at continuity-preserving remapping of genome segments between assemblies and provides features such as approximate locus conversion, automated batch processing and comprehensive logging to facilitate processing of datasets containing large numbers of structural genome variation data.

## Introduction

The first draft version of human genome was published in 2001 ^1^. In subsequent years, several new editions were released to perfect the quality of the genome assembly. The current version of human genome (GRCh38, UCSC hg38) was made available in 2013, with the latest revision (Grch38.p12) still containing more than 10 million unplaced bases ( see NCBI website). Over the years, large numbers of genomic studies have been performed, generating data mapped to different versions of the reference genome. However, when performing genome analyses integrating data from multiple resources, it is imperative to convert all data to the same genomic coordinate system.

Two general methodologies are used for conversion between coordinates from different genome assemblies. The first approach is to re-align the original sequence data to the target assembly. This method could provide the best result but is very time-consuming, and is not possible if the original sequence data is not available or does not consist of direct sequences (i.e. segmentation of array based data). Another approach is to convert the coordinates of genome data between assemblies by using a mapping file. This method, although bearing a side effect of minor information loss, for most applications provides a good balance between performance and accuracy.

Currently, three tools are in widespread use for the conversion between genome assemblies by coordinates: *liftOver* from University of California, Santa Cruz (referred as *UCSC liftOver* in the following article) ^2^; *CrossMap* from Zhao ^3^; and *Remap* from NCBI ^4^. The *UCSC liftOver* tool exists in two flavours, both as web service and command line utility. It offers the most comprehensive selection of assemblies for different organisms with the capability to convert between many of them. *CrossMap* has the unique functionality to convert files in BAM/SAM or BigWig format. It generates almost identical results as *UCSC liftOver*, but is not optimised for converting genome coordinates between species. *Remap* provides for each organism a comprehensive list of major assemblies and the corresponding sub-versions. It can also perform cross species mapping, however, with only a limited number of organisms.

**Figure 1.**
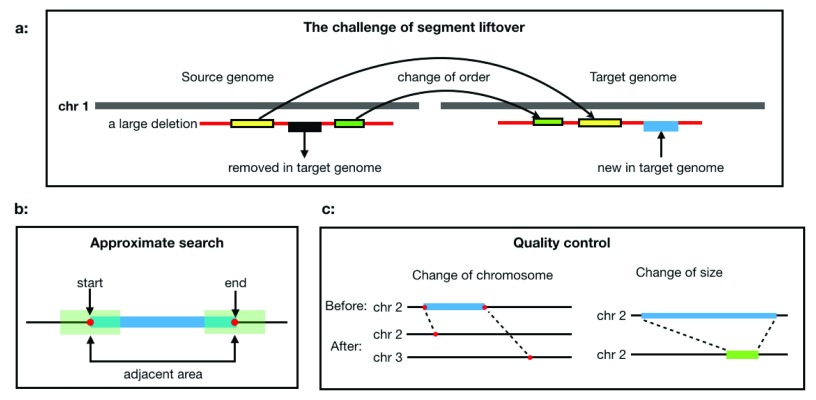
The challenge of segment liftover: ( **a**) When lifting a segment to another assembly, the landscape of the segment may be affected by indels and copy number variations, but the overall span of the segment does not change significantly. ( **b**) When the end positions cannot be converted by the *UCSC liftOver*, the nearby regions will be searched for convertible positions as approximation. ( **c**) Quality control checks for changes of chromosome or size to make sure the segment is converted properly.

All those tools are efficient in coordinate conversion and provide almost identical results. However, as shown in Figure 1a, challenges arise when dealing with genome segments that are not continuous anymore in the target assembly; there, these three tools take on different strategies. *CrossMap* and *UCSC liftOver* break the segment into smaller segments and map them to different locations. *Remap* keeps the integrity of the segment and maps the span to the target assembly.

In research such as analysis of copy number variation (CNV) data, where the quantitative representation of a genomic range takes precedence over base-specific representation, the integrity of a continuous segment indicates the proper conversion between assemblies, but may not be a direct outcome of current re-mapping approaches. Although *Remap* can convert contiguous segments, it only provides web service with submission limits, which is difficult to use for large scale or pipelined applications. The limitation to single input files is a general limitation of those tools, which precludes their direct use in comparative studies which may require to work with hundreds or thousands of files, as indicated through our own projects and requests from the research community.

In this article, we introduce *segment_liftover*, a tool to perform an integrity-preserving conversion of genomic segments data between genome assemblies. It features two major functional additions over existing tools: First, re-conversion by locus approximation, in instances where a precise conversion of genomic positions fails; and second, the capability to handle any number of files and optional integration into data processing pipelines with supporting features such as automatic file traversal, interruption resumption and detailed logging.

## Methods

### Implementation

*segment_liftover* can convert both probe files and segment files at the same time or in separate runs. It starts from a structured directory or a list of files, then traverses and converts all files meeting the specified name pattern, and finally outputs to a designated directory. To convert a probe file, *segment_liftover* will first use the *UCSC liftOver* to convert the file, then apply an approximate conversion on probes that the *UCSC liftOver* failed to convert. To convert a segment file, *segment_liftover* will use the *UCSC liftOver* to convert the *start* and *end* positions of segments. A successful segment conversion needs to satisfy the following four criteria:

*position _new_start_* ≠ ∅                                                       (1)

*position _new_end_* ≠ ∅                                                       (2)

*chromosome _new_start_* = *chromosome _new_end_*                             (3)

$$\frac{1}{\beta }\;<\;\frac{length(old\_segment)}{length(new\_segment)}\;<\beta \;(4)$$

Where *β* controls the threshold of the length ratio and the default value is set to 2. If criteria (1) or (2) fails, *segment_liftover* will apply an approximate conversion; if the conversion still fails, it is reported as unconvertible. If criteria (3) or (4) fails, the conversion is reported as rejected ( Figure 1c). The reason of failure is recorded in log files.

When a position cannot be converted by the *UCSC liftOver*, *segment_liftover* will attempt an approximate conversion and try to find a convertible position in the adjacency ( Figure 1b). The range and the resolution of the search is defined by parameters *–range* and *–step_size*, respectively.

### Operation

**Figure 2.**
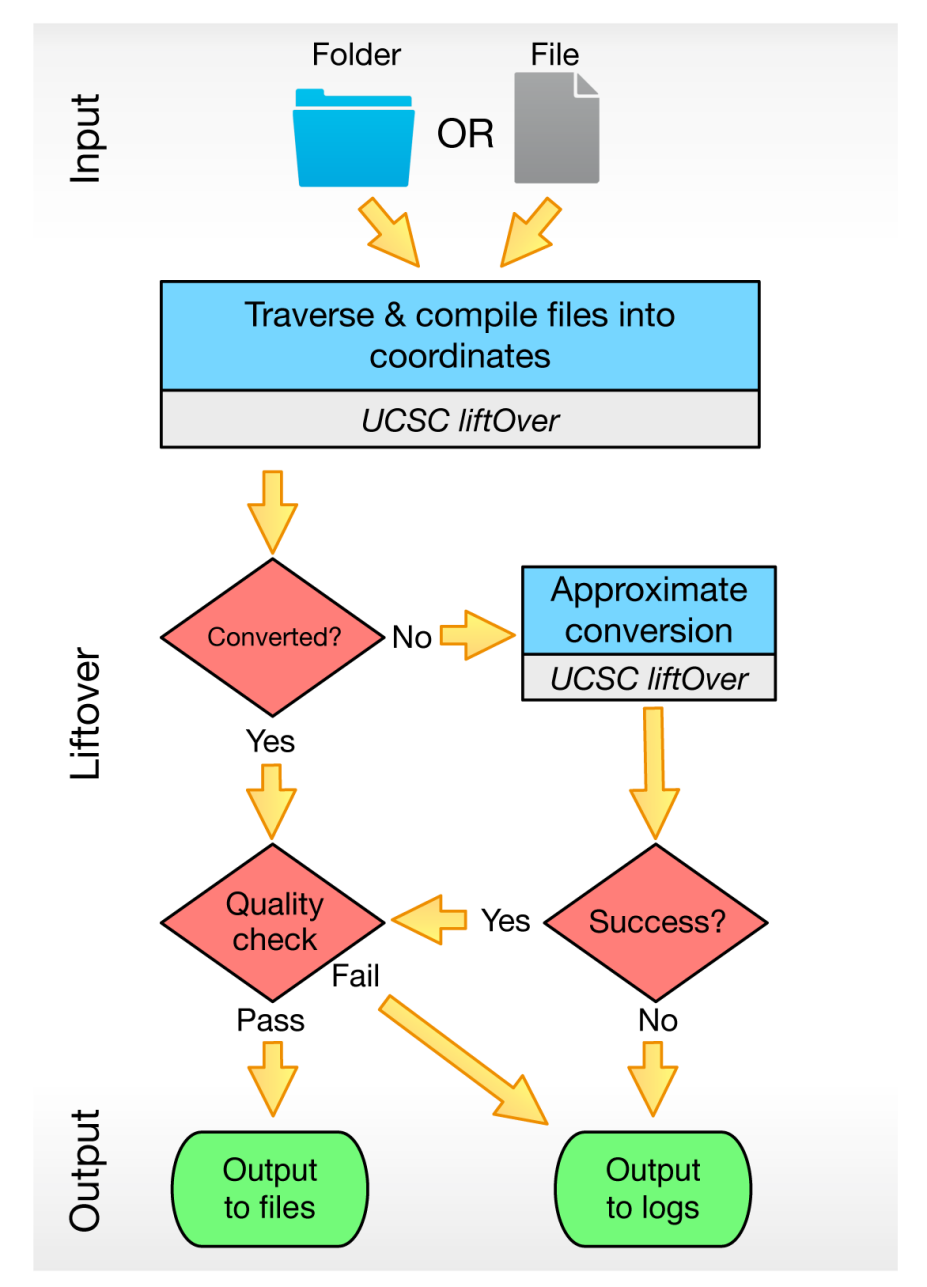
The workflow of *segment_liftover*: (1) It can take either a folder or a file containing the list of files as the input. (2) It will try to convert by approximation when *UCSC liftOver* fails to convert a coordinate. (3) The directory structure will be kept in the output folder and detailed log files are also available.

The *segment_liftover* tool is implemented in Python. The package is available for both Linux and OSX. It requires a minimum of 2G memory and the capacity of running Python 3. We recommend an installation using *pip* in a Python virtual environment. *segment_liftover* requires and depends on the *UCSC liftOver* program. A chain file, which provides alignments from source to target assembly, is also required. The chain files between common human assemblies (hg18, hg19 and hg38) are included in the program package. Chain files of other species and assemblies are available from the USCS Genome Browser. Figure 2 illustrates the work-flow of *segment_liftover*.

## Use cases

In this section, we provide two examples of using *segment_liftover* to convert probes and segments, respectively. The two examples are part of the pipeline which updates the *arrayMap* database, a reference resource of somatic genome copy number variations in cancer ^5^, from human genome assembly hg19 to hg38.

### Converting arrayMap data from hg19 to hg38

In the first example, we converted 44,632 probe files and 44,471 segment files from hg19 to hg38. The probe data were generated from nine Affymetrix genotyping array platforms, which currently only support annotations for hg19. Circular binary segmentation (CBS) analysis (DNAcopy R-package) was used to infer copy number segments from log2 values of probes. The final segment files contain a list of genomic regions separated by their copy number values ^6^. We ran the *segment_liftover* tool on a 12-core, 128GB RAM machine with 8 parallel processes. It took 42 hours to convert 44,632 probe files with 5.5 billion probe positions and 40 minutes to convert 44,471 segment files with 4.8 million segments.

**Figure 3.**
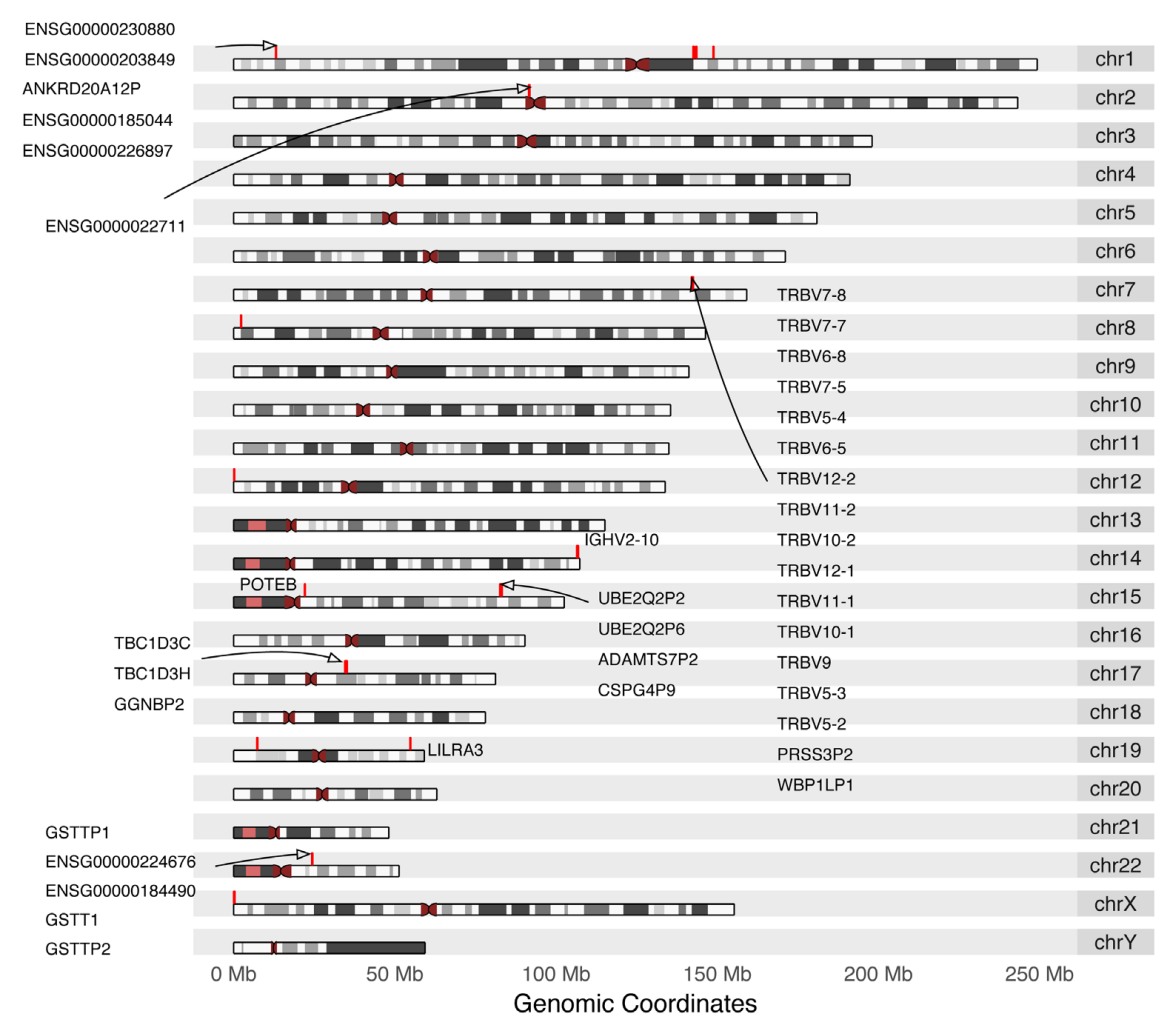
Genomic regions of unconvertible probe positions from human genome hg19. Unconvertible positions are marked red on the karyogram, annotated with HGNC symbol or ENSEMBL gene ID (if HGNC not available), retrieved from biomaRt_2.30.0.

Overall, more than 99.99% of probes and more than 99% of segments could be directly converted from hg19 to hg38 ( Table 1). As conversion of segments is more complicated and involves a quality control procedure to ensure the meaningfulness of the segment, it is expected to have a higher number of unconvertible segments than probes. As shown in Figure 3, the unconvertible regions are mainly around telomeres, centromeres, or other gene-sparse locations. In total, 38 genetic elements were found to be affected by the conversion (description in Supplementary Table 1).

**Table 1.** Conversion results: *Directly converted* is the sum of successful conversion from *UCSC liftOver*; *approximately converted* is the sum of successful approximate conversion; *converted but rejected* is the sum of all rejections from quality control; *unconvertible* is the sum of everything that can not be converted at all.

	probes	segments
directly converted	5,515,432,156	4,800,078
approximately converted	373,788	14,707
converted but rejected	953,624	41,201
unconvertible	10,914	1,064

### Comparison of different conversion strategies

**Figure 4.**
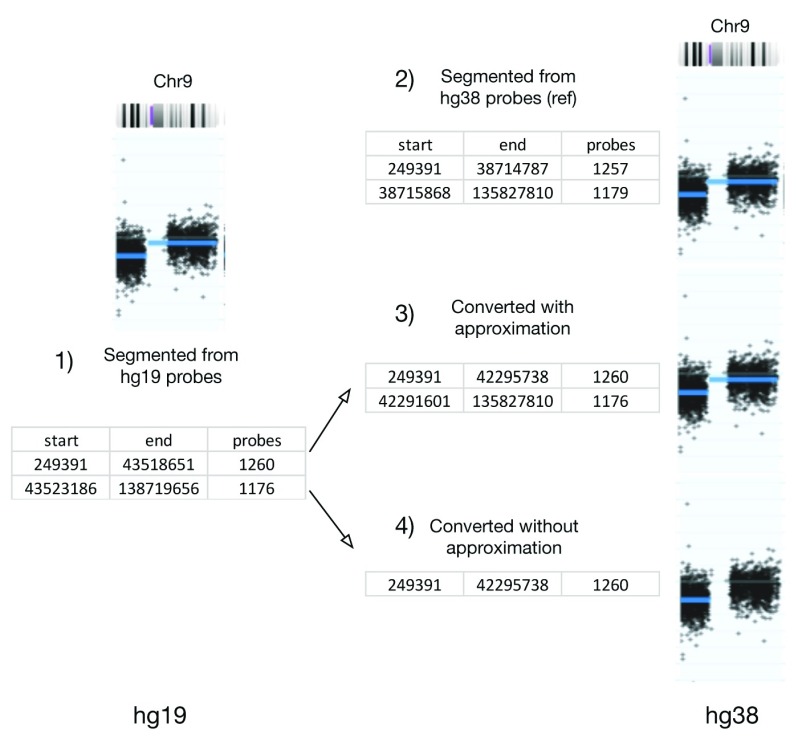
Chromosome 9 from GSM276858 with probe and segment data from hg19 coordinate(left) and hg38(right). Segments directly processed from hg38 probes are used as reference (top right). hg19 segments converted with approximation (middle right) and without approximation (bottom right) are used for comparison.

In the second example, we compared the performance of different conversion strategies using 1,000 samples randomly drawn from the first example ( Table 2). Copy number segments were generated using four different strategies ( Figure 4): (1) segments in hg19, which were generated directly from probes in hg19; (2) segments in hg38, which were generated from probes converted from hg19 to hg38 using *segment_liftover*; (3) segments in hg38, which were converted from hg19 to hg38 using *segment_liftover* with approximate conversion; (4) segments in hg38, which were converted from hg19 to hg38 using *segment_liftover* without approximate conversion. Strategy (1) is the standard procedure of copy number segment calling, and is used a the basic reference to compare with (2). Strategy (2) converts the raw data (probes) to hg38, then follows the same procedure as in (1). It is used as a reference for (3) and (4) to compare with. Strategies (3) and (4) convert segments (results from (1)) from hg19 to hg38, the difference is whether approximate conversion is used.

**Table 2.** Number of samples from nine platforms in use case examples.

	all	1000 samples
CytoScanHD_Array	2963	73
CytoScan750K_Array	173	2
Mapping50K_Hind240	2699	58
Mapping50K_Xba240	3303	72
Mapping10K_Xba142	912	19
Mapping250K_Nsp	9738	223
Mapping250K_Sty	7561	184
GenomeWideSNP_6	16570	359
GenomeWideSNP_5	552	10

Table 3 shows the comparison of conversion results in average between (3), (4) and (2) (complete table in Supplementary Table 2). Exact segment matches are categorized as "perfect"; "minor difference" is defined the same as condition (3) of quality control; the rest of the segments are categorized as "significant difference"; "sum" is average number of segments per sample. By comparing the sum of *reference hg19*, *reference hg38* and *approximation*, it shows that the conversion result is very close to the result of the standard pipeline. The difference between converted and generated sums is much smaller than the difference between two generated sums of different genome versions. On average, the approximate conversion could rescue one additional segment per file. Finally, we zoom into a specific example on chromosome 9 in GSM276858 (illustrated in Figure 4). Because of the removal of probes from hg19 to hg38, the second segment will be lost without approximate conversion.

**Table 3.** Number of segments with or without approximation on average.

	perfect	minor difference	significant difference	sum
reference hg19	*na*	*na*	*na*	218.42
reference hg38	*na*	*na*	*na*	215.04
approximation	198.18	6.24	10.25	214.67
no approximation	198.18	5.23	10.01	213.42

The two examples above demonstrated the efficiency and effectiveness of *segment_liftover* in processing large number of probe and segment files. It can provide conversion results that are similar to results generated from the standard pipeline. Moreover, with approximate conversion, the number of properly converted segments is slightly increased. In general, *segment_liftover* is able to provide reliable conversions and the ease of use.

## Summary

Translation between genome versions of sequencing data is a tedious but crucial task in bioinformatics. With the functionalities of automated batching, approximate conversion and segment conversion, *segment_liftover* can dramatically reduce the complexity and workload of such data processing. Furthermore, *segment_liftover*’s detailed logs of execution result provide an easy and clear foundation for follow up analysis.

## Software availability

1. pip version: https://pypi.python.org/pypi/segment-liftover
2. Latest source code: https://github.com/baudisgroup/segment-liftover
3. Archived source code as at time of publication: https://dx.doi.org/10.5281/zenodo.1186803 ^7^
4. Software license: MIT

## Supplementary material

Supplementary Table 1 : genetic elements affected by the conversion The 38 genetic elements that are affected by the segment_liftover conversion from hg19 to hg38.

Supplementary Table 2 : complete conversion results of different strategies A complete list of conversion results of comparing different conversion strategies using 1000 random samples.

## References

[1] Kent WJ Haussler D: Assembly of the working draft of the human genome with GigAssembler. *Genome Res.* 2001;11(9):1541–1548. 10.1101/gr.183201 11544197 PMC311095

[2] Kuhn RM Haussler D Kent WJ: The UCSC genome browser and associated tools. *Brief Bioinform.* 2013;14(2):144–161. 10.1093/bib/bbs038 22908213 PMC3603215

[3] Zhao H Sun Z Wang J: Crossmap: a versatile tool for coordinate conversion between genome assemblies. *Bioinformatics.* 2014;30(7):1006–1007. 10.1093/bioinformatics/btt730 24351709 PMC3967108

[4] NCBI Resource Coordinators: Database resources of the National Center for Biotechnology Information. *Nucleic Acids Res.* 2016;44(D1):D7–D19. 10.1093/nar/gkv1290 26615191 PMC4702911

[5] Cai H Gupta S Rath P: arrayMap 2014: an updated cancer genome resource. *Nucleic Acids Res.* 2015;43(Database issue):D825–D830. 10.1093/nar/gku1123 25428357 PMC4383937

[6] Olshen AB Venkatraman ES Lucito R: Circular binary segmentation for the analysis of array-based DNA copy number data. *Biostatistics.* 2004;5(4):557–572. 10.1093/biostatistics/kxh008 15475419

[7] Gao B Baudis M: baudisgroup/segment-liftover: First public version (Version 0.948). *Zenodo.* 2018 Data Source

