# Supplementary material for: segment_liftover : a Python tool to convert segments between genome assemblies
Source: F1000Res. 2018 Jun 8;7:319. Originally published 2018 Mar 14. [Version 2] doi: 10.12688/f1000research.14148.2 (PMC5998006; doi:10.12688/f1000research.14148.2)
Supplement: Peer review history

# Peer review: Referee response for version 2

**DOI:** 10.5256/f1000research.16527.r34839

**Published:** 2018-06-08

**Authors:** Ryan K. Dale

**Affiliations:** 1 Laboratory of Cellular and Developmental Biology, National Institute of Diabetes and Digestive and Kidney Diseases, National Institutes of Health, Bethesda, MD, USA

The revisions address my concerns -- and the new demo mode is especially useful.

I have read this submission. I believe that I have an appropriate level of expertise to confirm that it is of an acceptable scientific standard.

# Peer review: Referee response for version 1

**DOI:** 10.5256/f1000research.15390.r32847

**Published:** 2018-04-17

**Authors:** Ryan K. Dale

**Affiliations:** 1 Laboratory of Cellular and Developmental Biology, National Institute of Diabetes and Digestive and Kidney Diseases, National Institutes of Health, Bethesda, MD, USA

The authors describe how using existing genome coordinate conversion tools can be problematic on large domains in which continuity of the converted region is preferred over precision of the coordinate conversion. They describe a method that iteratively retries the conversion and applies QC criteria to the original and iterative conversions. The method is implemented using a Python package that wraps the UCSC liftover tool and performs the iterative remapping and QC steps, and provides this functionality over directories of files instead of on a per-file basis.

 To make it scientifically sound there should be a clear example in the documentation that works (details below). I have additional concerns and suggestions, particularly about the code, that I hope will improve the paper and the tool.

 Major comments:

 - Figure 3 is difficult to interpret as-is because some probes are double-counted across bars. I think a better approach would be to have a stacked bar for which the full height indicates the total number of probes that can be subset into converted and not-converted. These can be further subset by colors or by an adjacent stacked bar to indicate the proportion of converted that were directly converted or approximately converted, and the proportion of QC-failed.

 - I see in the code that there are some human-specific hard-coded conversions between chr23/24 and chrX/Y. This may cause unexpected results when running on other genomes. One solution would be to run a check on the chromosomes observed in the chainfile to see if it looks like a human liftover before doing the 23/24 -> X/Y conversions.

 - Currently the liftOver executable is required to be in the working directory or manually specified with the "--liftover" arg. A friendlier solution would be to expect liftOver to be found on the PATH variable (while also keeping the ability to explicitly specify if needed). This avoids having to symlink the executable to the working directory or requiring system-dependent command-line arguments.

 - A "logs" directory is created in the working directory, even just after running "segment_liftover --help". It would be nice to defer creating this directory until it's actually needed. This also means that running parallel jobs on a cluster from the same directory will clobber the log files, making debugging difficult. It would be better to expose log directory configuration on the CLI and/or put the logs directory as a subdirectory of the output directory by default.

 - the "tmp" directory behaves the same way (clutters the working directory; clobbers files when running in parallel). Furthermore it's not cleaned up after running. Better would be to use Python's tempfile module, which respects the environment's TMPDIR variable and is periodically automatically cleaned.

 - Example data in the correct directory structure should be provided, and a single command to run that data should be provided. That way the user can easily verify that everything is installed correctly. As it is, I had to create the directory structure and paste the example segments content from the README into a new file in that directory. That allowed segment_liftover to run, but then it failed on the example segments data in the README. Digging around in the logs, in general.log there appears to be an issue with int vs str expectations when loading as pandas.DataFrame (this is with pandas v0.22.0).

 - Are there requirements for chromosome names in segment files or probe files? The examples use integer chromosome names, but liftOver chainfiles use UCSC "chr" chromosome names. It appears that using an input file with "chr" names fails, again from expectations about int vs str columns in pandas. It would be good to describe what kind of chromosome names are supported (or link to a segments file specification if there is one).

 Minor comment:  Typo in text, "tensor" -> "tens or", and various typos in help text when running segment_liftover

I have read this submission. I believe that I have an appropriate level of expertise to confirm that it is of an acceptable scientific standard, however I have significant reservations, as outlined above.

# Peer review: Referee response for version 1

**DOI:** 10.5256/f1000research.15390.r31947

**Published:** 2018-03-26

**Authors:** Oscar Krijgsman

**Affiliations:** 1 Division of Molecular Oncology and Immunology, Netherlands Cancer Institute, Amsterdam, Netherlands

This manuscript discusses a new tool developed by the authors that improves the ‘liftover’ between genomic builds. “Segment_liftover” is built on the already available liftover tool from UCSC with the novelty that it the tool implements a better conversion between genomic builds for stretches of the genome (segments) like in for example DNA copy number analyses. In addition, the tool provides options to perform the analysis in batches and provides log files of execution and results. The tool, as available on github, provides ample documentation and examples to run without experiencing too many difficulties.

 The authors provide an interesting new tool that shows an improvement over existing tools. In general, the manuscript is clear and concise. However, a few minor points need to be addressed to improve the interpretation of the results described in the manuscript.

  

- The second use case is basically a direct comparison between UCSC liftover and the newly developed segment_liftover tool. However, this is not explicitly mentioned in the text. In the current version it is not evident that you did the nice/required comparison with a currently available tool. Furthermore, a little more explanation on the 4 different strategies would benefit the reader.
- Figure 3 does not convincingly convey its message. For example, it is not clear from the figure that the percentage of unconvertible segments is higher that probes although properly mentioned in the text. But also, the difference between the first bars is over a factor 1000. I understand the choice of a log10 scale but the authors might consider a different way of showing this data (table? Add percentages or number?).

I have read this submission. I believe that I have an appropriate level of expertise to confirm that it is of an acceptable scientific standard.
